# Epigenomic signature of major congenital heart defects in newborns with Down syndrome

**DOI:** 10.1186/s40246-023-00540-1

**Published:** 2023-10-06

**Authors:** Julia S. Mouat, Shaobo Li, Swe Swe Myint, Benjamin I. Laufer, Philip J. Lupo, Jeremy M. Schraw, John P. Woodhouse, Adam J. de Smith, Janine M. LaSalle

**Affiliations:** 1 Department of Medical Microbiology and Immunology, School of Medicine, University of California, Davis, CA USA; 2 Perinatal Origins of Disparities Center, University of California, Davis, CA USA; 3 Genome Center, University of California, Davis, CA USA; 4 MIND Institute, University of California, Davis, CA USA; 5 Center for Genetic Epidemiology, Department of Population and Public Health Sciences, Keck School of Medicine, University of Southern California, Los Angeles, CA USA; 6 Division of Hematology-Oncology, Department of Pediatrics, Baylor College of Medicine, Houston, TX USA

**Keywords:** Down syndrome, Congenital heart defect, Newborn dried blood spot, DNA methylation, Whole-genome bisulfite sequencing, Epigenetics, Epigenome-wide association study, Differentially methylated regions, nRBC, Hypomethylation

## Abstract

**Background:**

Congenital heart defects (CHDs) affect approximately half of individuals with Down syndrome (DS), but the molecular reasons for incomplete penetrance are unknown. Previous studies have largely focused on identifying genetic risk factors associated with CHDs in individuals with DS, but comprehensive studies of the contribution of epigenetic marks are lacking. We aimed to identify and characterize DNA methylation differences from newborn dried blood spots (NDBS) of DS individuals with major CHDs compared to DS individuals without CHDs.

**Methods:**

We used the Illumina EPIC array and whole-genome bisulfite sequencing (WGBS) to quantitate DNA methylation for 86 NDBS samples from the California Biobank Program: (1) 45 DS-CHD (27 female, 18 male) and (2) 41 DS non-CHD (27 female, 14 male). We analyzed global CpG methylation and identified differentially methylated regions (DMRs) in DS-CHD versus DS non-CHD comparisons (both sex-combined and sex-stratified) corrected for sex, age of blood collection, and cell-type proportions. CHD DMRs were analyzed for enrichment in CpG and genic contexts, chromatin states, and histone modifications by genomic coordinates and for gene ontology enrichment by gene mapping. DMRs were also tested in a replication dataset and compared to methylation levels in DS versus typical development (TD) WGBS NDBS samples.

**Results:**

We found global CpG hypomethylation in DS-CHD males compared to DS non-CHD males, which was attributable to elevated levels of nucleated red blood cells and not seen in females. At a regional level, we identified 58, 341, and 3938 CHD-associated DMRs in the Sex Combined, Females Only, and Males Only groups, respectively, and used machine learning algorithms to select 19 Males Only loci that could distinguish CHD from non-CHD. DMRs in all comparisons were enriched for gene exons, CpG islands, and bivalent chromatin and mapped to genes enriched for terms related to cardiac and immune functions. Lastly, a greater percentage of CHD-associated DMRs than background regions were differentially methylated in DS versus TD samples.

**Conclusions:**

A sex-specific signature of DNA methylation was detected in NDBS of DS-CHD compared to DS non-CHD individuals. This supports the hypothesis that epigenetics can reflect the variability of phenotypes in DS, particularly CHDs.

**Supplementary Information:**

The online version contains supplementary material available at 10.1186/s40246-023-00540-1.

## Introduction

Down syndrome (DS) is a set of distinct clinical features that result from trisomy 21, the most common autosomal aneuploidy across live births. Clinical characteristics of DS vary across individuals but include intellectual disability, short stature, muscle hypotonia, atlantoaxial instability, reduced neuronal density, cerebellar hypoplasia, and congenital heart defects (CHDs) [1]. CHDs affect ~ 50% of newborns of both sexes with DS [2–5] despite their diagnosis in only ~ 1% of newborns without DS [6]. The most frequently diagnosed CHD in children with DS is an atrioventricular septal defect (AVSD), a condition characterized by a large hole in the heart due to improper development of the endocardial cushion. Many cases of DS-CHDs, particularly AVSD, are diagnosed in utero by ultrasound, but others are not diagnosed until after birth following obvious symptoms or an echocardiogram and often require surgery.

The mechanisms influencing the development of CHDs among individuals with DS are not clear. Studies of partial trisomy 21 patients have pinpointed critical regions on chromosome 21, including the Down syndrome cell adhesion molecule (*DSCAM*) gene, that appear to underlie CHD development [7], but these have not addressed the incomplete penetrance among individuals with complete trisomy 21. Additionally, genome-wide association studies and candidate-gene approaches have identified variants on chromosomes throughout the genome that are associated with CHDs in DS [8–13]. However, these genetic variants do not sufficiently explain CHD risk among those with DS.

Another molecular driver or biomarker of CHD risk in children with DS may be epigenetic mechanisms such as DNA methylation. Increasing evidence has shown epigenetic alterations and gene-environment interactions to be involved in the pathogenesis of non-syndromic CHDs [14, 15], but comprehensive studies of genome-wide DNA methylation variation associated with DS-CHD are lacking. We previously used whole-genome bisulfite sequencing (WGBS) of newborn dried blood spots (NDBS) to examine methylation profiles in 11 DS-CHD compared to 10 DS non-CHD samples, as part of a larger DS versus typical development (TD) study [16]. There were 1588 nominally significant (*p* < 0.05) differentially methylated regions (DMRs) (35% hypermethylated, 65% hypomethylated) distinguishing DS-CHD from DS non-CHD, and these regions were enriched for terms related to the heart, as well as neurodevelopment and metabolism [16]. These promising but preliminary results suggesting an epigenomic signature of CHD within DS led us to conduct the present study.

This current study used WGBS of NDBS obtained from the California Biobank Program among 86 DS individuals with and without major CHDs to identify specific loci, biological pathways, and genic contexts that are associated with risk for CHDs in the DS population. Very few studies have conducted WGBS on NDBS, a sample source that is accessible, widely banked, reflective of the intrauterine period, and informative regarding dysregulation in other tissues, including the brain and the heart [16]. In contrast to reduced representation methods such as arrays, this WGBS study provides insight to the entire DS-CHD epigenome, particularly because regional methylation smoothing approaches increase confidence over regions with relatively low coverage. Additionally, our study investigates similarities/differences in molecular signatures of DS-CHD in males compared to females, as well as DS-CHD compared to DS (versus TD). Our findings showed sex-specific global and region-specific changes to methylation that may serve as biomarkers and/or be functionally important in the development of CHDs in individuals with DS.

## Methods

### Study populations and DNA extraction from NDBS

This study was approved by Institutional Review Boards at the California Health and Human Services Agency, University of Southern California, and University of California, Davis. For the Discovery study, deidentified NDBS were obtained from 90 newborns with DS from the California Biobank Program (CBP, SIS request number 572), with a waiver of consent from the Committee for the Protection of Human Subjects of the State of California [17]. Demographic and birth-related data, including sex, race/ethnicity, birthweight, gestational age, and age of blood collection, were obtained from the CBP (Additional file 1: Tables S1 and S2). DS newborns with CHD or without CHD were identified via linkage between the California Department of Public Health Genetic Disease Screening Program and the California Birth Defects Monitoring Program (CBDMP). In brief, the CBDMP is a population-based surveillance program that covers ~ 30% of the births in California, including 10 counties, which are representative of the state’s population [18]. Birth defects diagnosis data from CBDMP for the 90 newborns were coded into “major birth defects” and “major heart defects” using guidelines from the National Birth Defects Prevention Network [6]. Major defects included AVSD and tetralogy of Fallot. We identified 46/90 newborns with a CHD, of which 44 were AVSDs, and 3 had tetralogy of Fallot. For this study, we focused on major heart defects and following sample quality control (QC) (described below) we included 45 DS with CHD (27 female, 18 male) and 41 DS without CHD (27 female, 14 male). DNA was extracted from one 4.7 mm card punch of each of the 90 NDBS, roughly 1.4 cm in diameter, with the Beckman Coulter GenFind V3 Reagent Kit (cat #C34880).

### Whole-genome bisulfite sequencing

All DNA samples were sonicated to ~ 350 bp with a peak power of 175, duty of 10%, 200 cycles/burst, and a time of 47 s. The sonicated DNA was cleaned and concentrated with Zymo gDNA clean and concentrator columns and eluted in 25 µl EB. Bisulfite conversion was performed with the Zymo EZ DNA Methylation Lightning Kit (cat #11-338) using ~ 35 ng of each sonicated sample. Libraries were prepared using the Swift ACCEL-NHS MethylSeq DNA Library Kit (cat #30096) with 7 cycles of PCR for normal-input samples and 11 cycles for low-input samples. Libraries were pooled, and a 0.85X SPRI cleanup was performed on 250 µl of the pool, eluted in 100 µl. The library pool (concentration of 3.63 ng/µl) was sequenced across 4 lanes of an Illumina NovaSeq 6000 S4 flow cell using 150 bp paired end reads.

FASTQ files for each sample were merged across lanes using FASTQ_Me [19] and aligned to the hg38 genome using CpG_Me [20] with the default parameters [21–24]. The alignment pipeline includes trimming adapters and correcting for methylation bias, screening for contaminating genomes, aligning to the reference genome, removing PCR duplicates, calculating coverage and insert size, and extracting CpG methylation to generate a cytosine report (CpG count matrix) and a QC report. Global methylation for each sample was calculated as the total number of methylated CpG counts divided by the total number of CpG counts from CpG count matrices. From the 90 samples sequenced, four samples were removed from analysis: two due to high levels of sequence duplication and two due to missing sample data.

### Genome-wide DNA methylation arrays

In addition to WGBS, existing DNA methylation data were available from NDBS for each sample from Illumina Infinium MethylationEPIC (EPIC) DNA methylation arrays [17]. In brief, DNA was isolated from a separate one-third portion of the NDBS, bisulfite conversion performed as above, and DNA samples were block-randomized (ensuring equivalent distribution of sex and race/ethnicity on each plate) for EPIC arrays [17]. QC of DNA methylation array data was conducted in R using “minfi,” “SeSAMe,” and “noob” packages, and trisomy 21 was confirmed from copy-number variation plots generated using the conumee R package [25], as described [17]. Global methylation for each sample is represented by the beta value, the ratio of the methylated probe intensity and the overall intensity [26]. As previously done [27], EPIC array beta values from all samples were correlated with WGBS global methylation values to assess consistency across platforms (see Sect. 2.5). DMRs associated with DS-CHDs were investigated using the ipDMR method with the ENmix R package [28].

### Cell-type estimation

To estimate nucleated cell proportions in NDBS samples, we used the EPIC array data to perform reference-based deconvolution using the Identifying Optimal Libraries (IDOL) algorithm [29]. Briefly, “estimateCellCounts2” function from the FlowSorted.Blood.EPIC R package [30] was used to estimate proportions of CD8 + T lymphocytes (CD8T), CD4 + T lymphocytes (CD4T), natural killer (NK) cells, B lymphocytes (B cell), monocytes, granulocytes, and nucleated red blood cells (nRBC), using cord blood cell reference samples included in the FlowSorted.CordBloodCombined.450 k R package [31].

### Sample trait analysis

Newborn sample traits of global CpG methylation, birthweight, gestational age of delivery, age of blood collection, race, ethnicity, and cell-type proportions were correlated using Pearson’s method with the Hmisc package v4.7.1 [32], and *p* values were adjusted by FDR (0.05 threshold) using the corr.test function in the psych package v2.2.9 [33] in R v4.1.3. DS-CHD versus DS non-CHD samples (sex-combined and sex-segregated) were tested for differences across sample traits using Welch’s unpaired variances *t* test with GraphPad Prism v9.4.1. Stepwise forward logistic regression was performed to determine the variables that best predicted CHD in each sex using the “glm (family = binomial)” function in R v4.1.3. Stepwise linear regression was performed to determine the variables that best predicted global CpG methylation in each sex using the lm function in R v4.1.3.

### DMR analysis from WGBS

DMRs for DS-CHD versus DS non-CHD in the WGBS data were called for Sex Combined, Females Only, and Males Only samples using DMRichR v1.7.3 [16] and R v4.1.0. Default parameters were used to identify DMRs containing at least 5 CpGs with at least a 5% methylation difference between groups, with each CpG requiring at least 1 × coverage in at least 75% of samples. These cutoffs were selected to leverage the genome-wide coverage of low-pass WGBS (~ 4 × coverage) by including a large proportion of total CpGs in the analyses (Additional file 2: Fig. S1) [34]. To increase confidence of putative DMRs despite a requirement of only 1 × coverage over each CpG, DMRichR implements a smoothing approach that gives greater weight to CpGs with higher coverage and infers the methylation levels of nearby CpGs that have lower coverage. Specifically, DMRichR uses bsseq [35] to extract methylation levels from cytosine reports and dmrseq [36] to identify DMRs. The dmrseq algorithm detects candidate regions whose smoothed pooled methylation proportion shows differences between groups and then assesses the significance of candidate regions through permutation testing of the pooled null distribution to calculate *p* values that are then FDR corrected to generate q-values [36].

In all three comparisons (Sex Combined, Females Only, and Males Only), we adjusted for sample traits that were correlated with global methylation (|*r*|> 0.2): age of blood collection and all cell types. Sex was additionally adjusted for in the Sex Combined analysis. Gestational age and birthweight met this cutoff in males, but not females, and were not corrected for because the effect of gestational age on DNA methylation has been found to mostly be due to nRBC proportion [37] and birthweight is largely dependent on gestational age. Sex chromosomes were included in Females Only and Males Only comparison but not the Sex Combined comparison. The sex of each sample was confirmed by the number of reads of sex chromosomes as previously described [16].

Principal component analysis (PCA) was performed using smoothed methylation values over the DMRs identified in each comparison to test for separation of CHD and non-CHD samples. Data were standardized so each variable had a mean of 0 and standard deviation of 1, and principal components were selected by parallel analysis from 1000 permutations using GraphPad Prism v9.4.1. The two principal components that explained the greatest variance in the data were selected for graphing and samples were color-coded by CHD and non-CHD. Sex specificity of the DMRs was tested by obtaining smoothed methylation values over DMRs from the Males Only comparison in female samples and over DMRs from the Females Only comparison in male samples, and PCA was performed as explained above.

Machine learning algorithms implemented through DMRichR were used to identify minimal DMRs for classifying samples as CHD or non-CHD [16]. Random forest algorithms from the Boruta package [38] and support vector machine algorithms from the sigFeature package [39] were used to build binary classification models and rank the DMRs by importance for the feature selection analyses. Minimal DMRs were selected as those that were identified in both lists and were in the top 1%.

DS-CHD DMRs from Sex Combined, Females Only, and Males Only comparisons were overlapped by genomic coordinates using rtracklayer v1.54.0 [40] and GenomicRanges v1.46.1 [41], and the Venn diagram was made with VennDiagram v1.7.3 [42] in R v4.1.3. The significance of the overlap between any two DMR comparisons was tested by permutation testing using the regioneR R package v1.32.0 [43]. The true overlap was compared to a null distribution of overlaps containing 10,000 length-matched random regions from the “universe” which we defined as the intersection of the total regions that were tested as potential DMRs for each comparison.

### Enrichment testing and gene ontology from WGBS DMRs

DMRs from all comparisons were tested for enrichment in chromosome location compared to background regions using the Database for Annotation, Visualization and Integrated Discovery (DAVID), 2021 version [44, 45]. DMRs were tested for enrichment in genic (promoter, 5’UTR, exon, intron, 3’UTR, downstream, intergenic) and CpG (island, shore, shelf, open sea) contexts compared to background regions using DMRichR [16]. The significance of genic and CpG annotations was tested using Fisher’s exact test and FDR correction. DMRs were mapped to genes on the hg38 genome using TxDb. Gene ontology enrichment was performed using rGREAT [46], with genomic coordinates of DMRs tested relative to background regions using the “oneClosest” rule.

### Replication of WGBS CHD DMRs in independent DS newborn study

DMRs were tested for replication in a previously published DS NDBS WGBS dataset with 10 non-CHD (2 female, 8 male) and 11 CHD (6 female, 5 male) individuals [16]. Unadjusted smoothed methylation values were calculated in replication dataset samples over DMR genomic coordinates from Sex Combined, Females Only, and Males Only comparisons using the “getMeth” function of the bsseq R package [35]. Unpaired *t* tests were calculated using the smoothed methylation values for replication CHD versus non-CHD samples, and *p* values were corrected by FDR using GraphPad Prism v9.4.1.

### Comparison of WGBS CHD DMRs and background regions with DS versus TD NDBS samples

DS-CHD DMRs and background regions were tested for overlap with DMRs associated with DS in a previous epigenome-wide association study that included 21 DS (8 female, 13 male) and 32 TD (16 female, 16 male) NDBS samples with WGBS data [16]. Unadjusted smoothed methylation values were calculated in replication dataset samples over DMR and background region genomic coordinates from Sex Combined, Females Only, and Males Only analyses using the “getMeth” function of the bsseq R package [35]. Unpaired *t* tests were calculated for DS versus TD using the smoothed methylation values of the replication dataset, and *p* values were corrected by the FDR method using GraphPad Prism v9.4.1. Potential differences between the proportions of DS-CHD DMRs and background regions that were significantly differentially methylated in DS versus TD or methylated in the same direction in DS versus TD as DS-CHD versus DS non-CHD were calculated using the *z* test for two population proportions.

## Results

### Sample traits were not different in DS-CHD cases compared to DS non-CHD controls

We quantitated DNA methylation by EPIC array and WGBS in DNA isolated from NDBS from 86 individuals with DS, 45 of whom also had a CHD. Overall, our cohort had more females [*n* = 54 (62.8%), CHD = 27, non-CHD = 27] than males [*n* = 32 (37.2%), CHD = 18, non-CHD = 14] as well as a higher proportion of Hispanic participants [*n* = 57 (63%)] and lower proportion of non-Hispanic white [*n* = 17 (19.8%)], non-Hispanic Asian [*n* = 8 (9.3%)], and non-Hispanic Black [*n* = 4 (4.7%)] participants when compared to California census data [47]. However, there were no significant differences for sex or race/ethnicity between DS-CHD and DS non-CHD newborns (Table 1) (Additional file 1: Table S3). In addition, birthweight, gestational age, and age at blood collection did not differ significantly between DS-CHD and DS non-CHD newborns (Table 1) (Additional file 1: Table S3).

**Table 1:** Sample traits in DS-CHD cases and DS non-CHD controls

	All samples (*n* = 86) Mean or *n* (SD or %)	DS-CHD (*n* = 45) Mean or *n* (SD or %)	DS non-CHD (*n* = 41) Mean or *n* (SD or %)
Global methylation	79.3 (3.9)	79.0 (4.3)	79.5 (3.4)
CD8T	0.05 (0.03)	0.05 (0.03)	0.05 (0.04)
CD4T	0.12 (0.08)	0.13 (0.09)	0.11 (0.07)
NK	0.03 (0.02)	0.03 (0.03)	0.03 (0.02)
B cells	0.007 (0.009)	0.006 (0.009)	0.007 (0.01)
Monocytes	0.07 (0.04)	0.07 (0.05)	0.07 (0.04)
Granulocytes	0.58 (0.21)	0.54 (0.23)	0.63 (0.17)
nRBCs	0.12 (0.23)	0.15 (0.27)	0.09 (0.19)
Birthweight (grams)^a^	3018 (633)	2953 (636)	3092 (630)
Gestational age (days)^b^	266 (17)	266 (16)	267 (18)
Age of blood collection (hours)	62 (57)	63 (49)	62 (65)
*Sex*			
Female	54 (62.8%)	27 (60%)	27 (65.9%)
Male	32 (37.2%)	18 (40%)	14 (34.1%)
*Race/ethnicity*			
Asian (non-Hispanic)	8 (9.3%)	6 (13.3%)	2 (4.9%)
Black (non-Hispanic)	4 (4.7%)	3 (6.7%)	1 (2.4%)
White (non-Hispanic)	17 (19.8%)	9 (20%)	8 (19.5%)
Hispanic	57 (66.3%)	27 (60%)	30 (73.2%)

^a^Missing data from 1 sample (DS non-CHD)

^b^Missing data from 5 samples (4 DS-CHD, 1 DS non-CHD)

The estimated cell-type proportions (CD8T, CD4T, NK, B cell, monocytes, granulocytes, nRBC) in newborn blood were highly variable across samples, particularly for nRBC and granulocyte proportions (Additional file 1: Table S2). All cell types were positively correlated with one another, except nRBCs which were negatively correlated with all other cell types (Additional files 1: Table S4, 2: Fig. S2). Most cell types (CD8T, CD4T, NK, monocytes, nRBC) were also significantly (unadjusted *p* < 0.05) correlated with age of blood collection, and all cell types were significantly correlated with WGBS global methylation levels, supporting the adjustment for cell types in our DMR analyses. Cell-type proportions did not differ significantly between DS-CHD and DS non-CHD newborns overall, or in sex-stratified comparisons (Table 1) (Additional file 1: Table S3).

### WGBS of newborn blood DNA detects global hypomethylation in DS-CHD males compared to DS non-CHD males due to elevated nRBC proportions

To assess the reproducibility of EPIC array and WGBS methylation quantitation, we examined global CpG methylation levels from the two platforms and found that EPIC array beta values (Additional file 1: Table S5) were lower than WGBS global methylation values across all samples, but very strongly correlated (*r *= 0.9716, *p* < 0.0001) (Additional file 2: Fig. S3), consistent with previous findings [27]. While a few samples had notably low global CpG methylation levels (< 70% from WGBS), these samples were not removed from analysis because their other QC metrics were acceptable and their corresponding array beta values were also low, suggesting it was not a technical error.

**Fig. 1:**
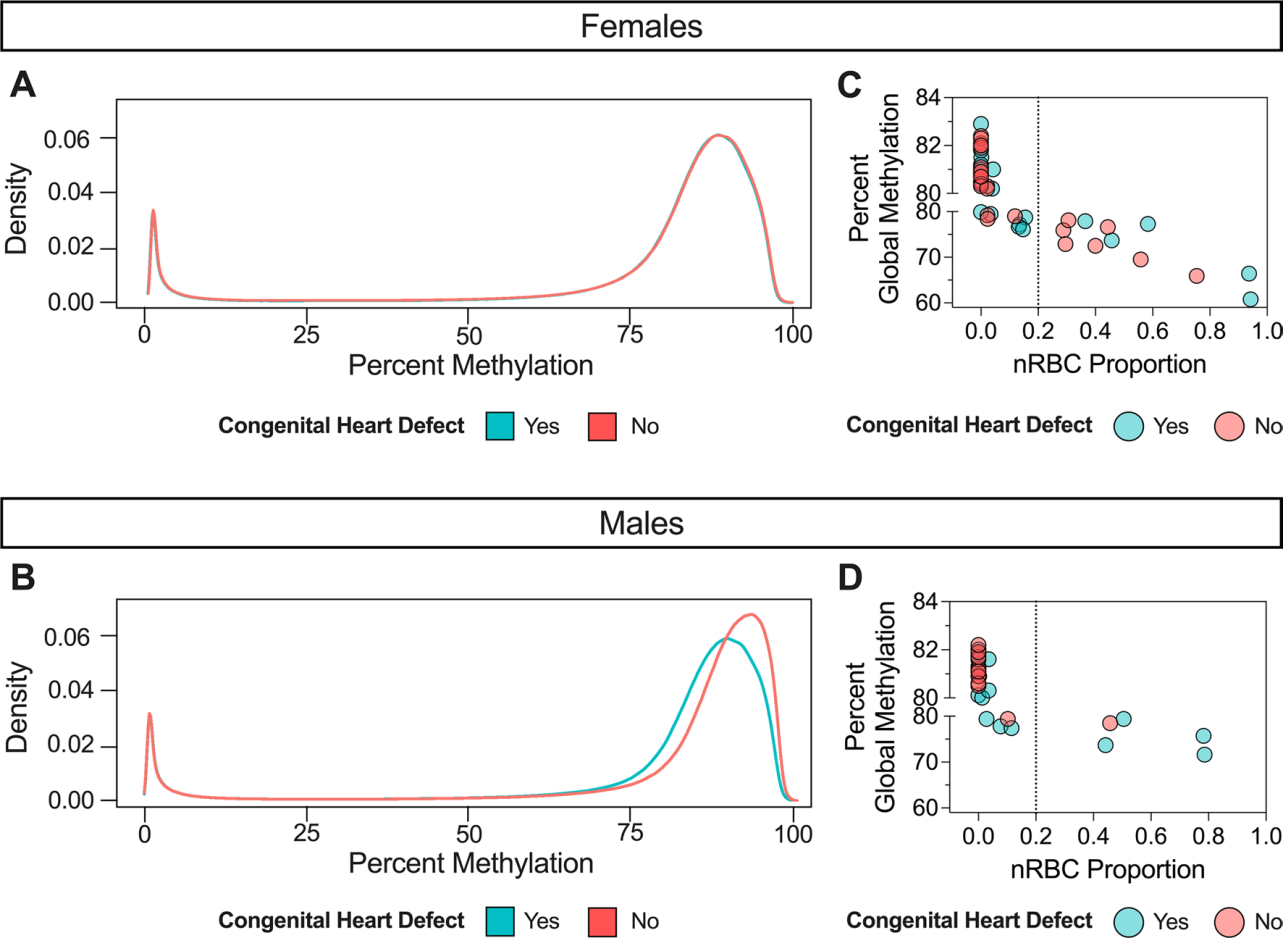
Global hypomethylation in DS-CHD males versus DS non-CHD males is driven by samples with high nRBC proportions. Density plot of average percent smoothed methylation in DS-CHD (Yes: blue) and DS non-CHD (No: red) in **A** females (note that red and blue lines are overlapping) and **B** males. Percent global methylation correlated with nRBC proportion in **C** females (Pearson’s *r *=  − 0.93, *p* = 2.16E−24) and **D** males (Pearson’s *r *=  − 0.84, *p *= 2.13E−9)

Using WGBS data, we first assessed whether global CpG methylation levels differed between DS-CHD and DS non-CHD newborns. There was no significant difference overall, but when stratified by sex we found significant hypomethylation in DS-CHD males compared with DS non-CHD males (unadjusted *p* < 0.05), a pattern that was not seen in females (Fig. 1A, B) (Additional files 1: Table S3, Fig S4). We confirmed by logistic regression that global methylation was the most predictive variable of CHD in males (*p* = 0.101), while CD4T cell proportion was the most predictive variable in females (*p* = 0.0681) (Additional file 1: Table S6). Because nRBCs are known to have lower methylation levels than other cell types in blood [48] and their proportion in blood samples varies widely across individuals [49] in negative association with global methylation [48, 50], we investigated the relationship between nRBC proportion and global methylation in our samples. In both females and males, nRBC proportion was significantly negatively correlated with global methylation levels (Fig. 1C, D) (Additional files 1: Table S4, Fig S2) and was the most predictive variable of global methylation in linear regression models (females *p* <  − 2E16, males *p* = 2.12E−9) (Additional file 1: Table S6). The presence of CHD predicted global methylation levels in males (*p* = 0.0618) much better than in females (*p* = 0.981), but addition of nRBC proportion as an adjustment covariate decreased the strength of this relationship (males, *p* = 0.237) (Additional file 1: Table S6).

While proportion of nRBCs in the nucleated cell population is typically very low, with a median of 0 for the estimated nRBC proportions across samples in our study, we identified 12 out of 32 male samples with nRBC proportions > 1%, of which 10 (83%) had a CHD (Supplemental Table S2) (Additional file 2: Fig. S4). In contrast, we identified 24/54 females with nRBC proportions > 1% of which only 50% (*n* = 12) had a CHD. In a sensitivity analysis, we removed five male samples that had notably high nRBC levels (> 20%) and corresponding low global methylation levels and saw that global methylation in DS-CHD versus DS non-CHD male samples were no longer significantly different (*p* = 0.1865) (Additional file 1: Table S3). Females showed high interindividual variation in global methylation levels and nRBC proportions in both CHD and non-CHD groups, while CHD males showed much more variation (similar to females) than non-CHD males (Additional file 2: Fig. S4).

### Sex-stratified DMRs distinguish DS-CHD from DS non-CHD samples better than sex-combined DMRs

Next, using WGBS data we investigated whether there were DMRs associated with DS-CHDs in Sex Combined, Females Only, and Males Only comparison groups to characterize both sex-specific and sex-independent patterns in DS-CHD methylation. We adjusted for confounding variables that were associated with global methylation (|*r*|> 0.2): age of blood collection and all cell-type proportions, as well as sex (specific for the Sex Combined comparison) (Additional files 1: Table S4, Fig. S2).

The Sex Combined comparison yielded 58 significant by permutation (*p* < 0.05) DMRs (Additional file 1: Table S7). In Females Only, we found 341 DMRs (Additional file 1: Table S8), whereas in Males Only we found 3938 DMRs (Fig. 3A) (Additional file 1: Table S9). Samples with low methylation levels across Males Only DMRs corresponded with those with low global methylation. In a sensitivity analysis excluding the five male samples with nRBC proportions > 20%, we identified 2474 Males Only DMRs (Additional file 1: Table S10).

**Fig. 2:**
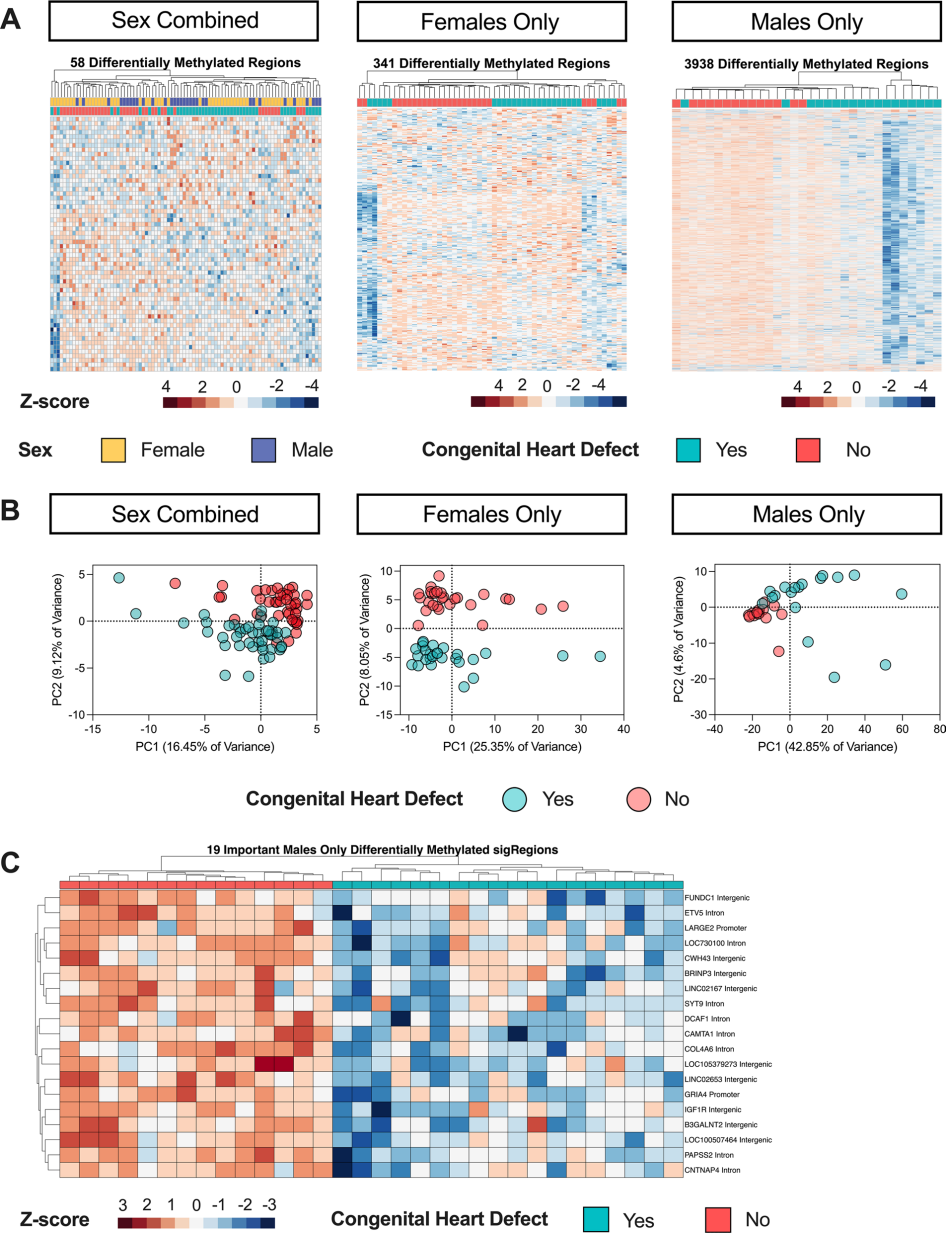
DMR profiles of CHD versus non-CHD in Sex Combined, Females Only, and Males Only comparisons within DS. **A** Heatmaps of nominally significant (*p* < 0.05) DMRs from DS-CHD versus DS non-CHD samples in Sex Combined, Females Only, and Males Only comparisons. All heatmaps show hierarchical clustering of Z-scores, which are the number of standard deviations from the mean of non-adjusted percent smoothed individual methylation values for each DMR. **B** PCA analysis using the smoothed methylation values of all DMRs from the Sex Combined and Females Only comparisons and the 1000 most significant DMRs in the Males Only comparison. **C** Hierarchical clustering heatmap of the machine learning feature selection analysis of the consensus DMRs from the Males Only comparison

DMR hierarchal clustering and principal component analysis (PCA) showed that CHD and non-CHD samples did not separate completely, although sex stratification improved the distinction (Fig. 2A, B). Using machine learning feature selection, we identified a minimal set of 19 Males Only DMRs that could distinguish CHD from non-CHD samples (Fig. 2C) (Additional file 1: Table S11). The five male samples with high nRBCs and low methylation across DMRs did not have outlier methylation values across the 19 minimal DMRs, showing that the most predictive DMRs were not driven by outliers. In the Males Only sensitivity analysis (with 5 samples with nRBC > 20% removed), 13 minimal DMRs distinguished CHD from non-CHD, with four overlapping with those from the Males Only minimal selection using all samples: *DCAF1, LARGE2, LOC105379273, SYT9* (Additional file 1: Table S11). In the other comparisons, 6 Sex Combined and 3 Females Only DMRs were identified by the feature selection but could not cleanly distinguish CHD from non-CHD samples (Additional files 1: Table S11, Fig. S5).

In the replication dataset of WGBS from NDBS of 21 children with DS, 11 with CHD (6 females, 5 males) and 10 without CHD (2 females, 8 males) [16], we found that 26 (46.4%) of the 56 Sex Combined DMRs that were covered in the replication study were methylated in the same direction in both groups (Additional file 1: Table S12), while 161/329 (48.9%) of Females Only (Additional file 1: Table S13) and 2229/3938 (56.7%) of Males Only DMRs (Additional file 1: Table S14) were methylated in the same direction. Few DMRs were significantly differentially methylated (unadjusted *p* < 0.05) in the replication dataset, with 2 Sex Combined, 9 Females Only, and 68 Males Only meeting this cutoff.

In DMR analysis using EPIC array data, there were no significant DMRs associated with DS-CHDs, likely due to the EPIC array only covering ~ 3% of CpGs covered by WGBS (data not shown).

### DS-CHD DMRs are sex-specific, with a small fraction overlapping across sexes

**Fig. 3:**
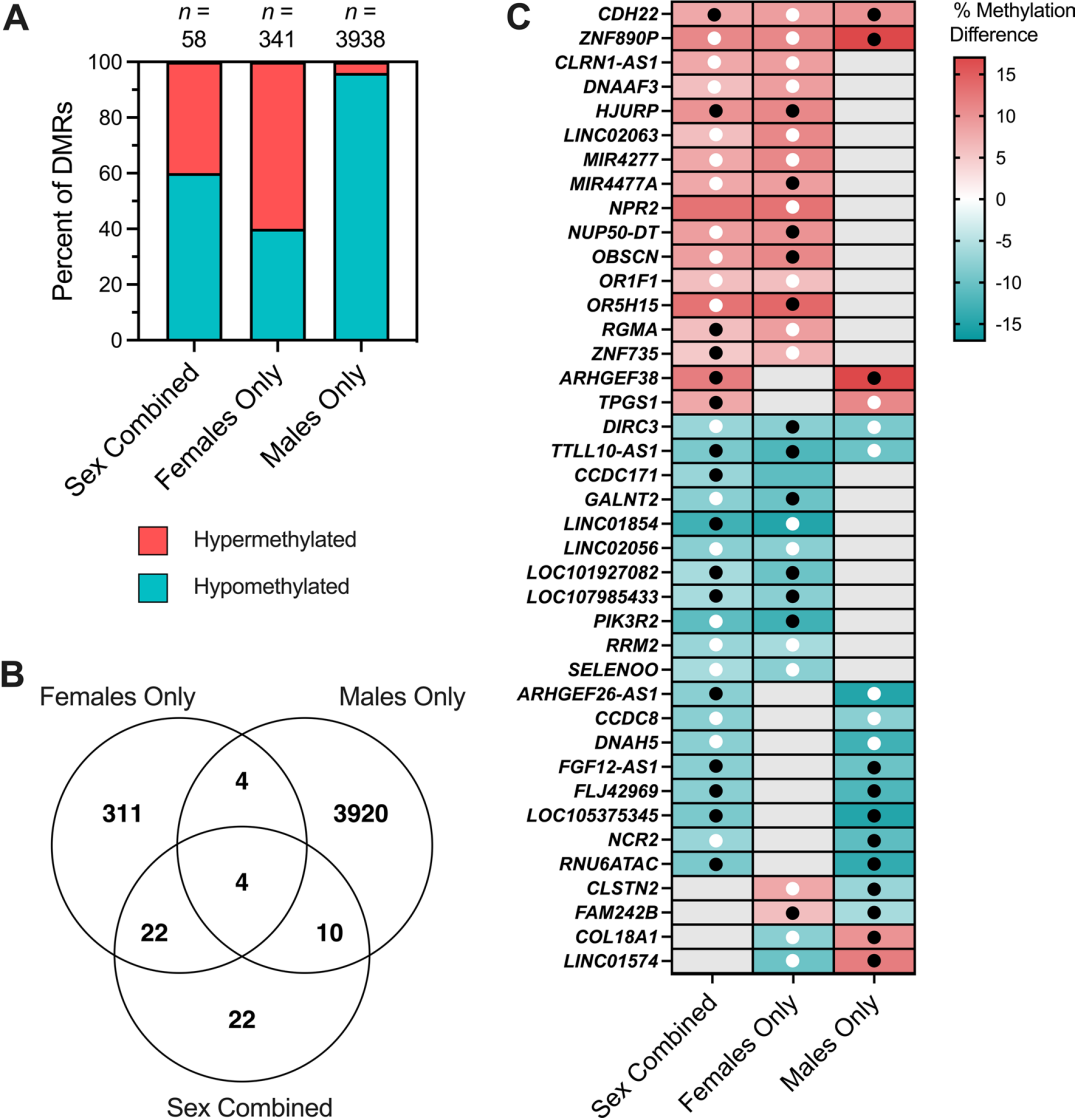
Overlapping CHD DMRs across Sex Combined, Female Only, and Male Only comparisons within DS. **A** The percent of DMRs which were hypermethylated versus hypomethylated in each of the three comparisons. **B** Venn diagram reflecting the numbers of unique and overlapping DMR genomic coordinates across the three comparisons. **C** DS-CHD DMRs which overlap in two or more comparisons mapped to genes. Red indicates hypermethylation in CHD compared to non-CHD, while blue represents hypomethylation, with stronger shades representing a greater percent methylation difference. Gray is used when a DMR was not called for that comparison. Black dots indicate methylation in the same direction in the discovery and replication datasets [10 non-CHD (2 female, 8 male) and 11 CHD (6 female, 5 male)], while white dots indicate methylation in the opposite direction in the two datasets. No dot means that the DMR genomic coordinates were not covered in the replication dataset

We next examined similarities and differences in DMRs across females and males. In the Sex Combined comparison, 60% of DMRs were hypomethylated in CHD compared to non-CHD samples, while 40% of Females Only DMRs and 96% of Males Only DMRs were hypomethylated (Fig. 3A). In our Males Only sensitivity analysis that removed samples with nRBC proportions > 20%, 82% of DMRs were hypomethylated. To test the sex specificity of Females Only and Males Only DMRs, we analyzed the smoothed methylation values over DMRs from the Males Only comparison in female samples and from the Females Only comparison in male samples and found that CHD and non-CHD samples did not separate by PCA (Additional file 2: Fig. S6). Females and males also did not separate by hierarchal clustering or PCA in the Sex Combined comparison (Fig. 2A) (Additional file 2: Fig. S7).

DMR genomic coordinates from all comparisons were then overlapped to identify sex-specific versus sex-independent regions. Four DMRs overlapped across all three comparisons, 26 across Sex Combined and Females Only comparisons (*p* < 9.99E−05), 14 across Sex Combined and Males Only comparisons (*p* < 9.99E−05), and 8 across Males Only and Females Only comparisons (*p* = 0.207) (Fig. 3B) (Additional file 1: Table S15). All overlapping DMRs between comparison groups were methylated in the same direction except for the 4 overlapping between Females Only and Males Only comparisons (but not the Sex Combined comparison), which showed methylation in opposite directions (Fig. 3C). The 4 DMRs identified in all three comparisons mapped to *CDH22, ZNF890P, DIRC3*, and *TTLL10-AS1* genes.

### DS-CHD DMRs are enriched for gene exons, CpG islands, and bivalent chromatin

**Fig. 4:**
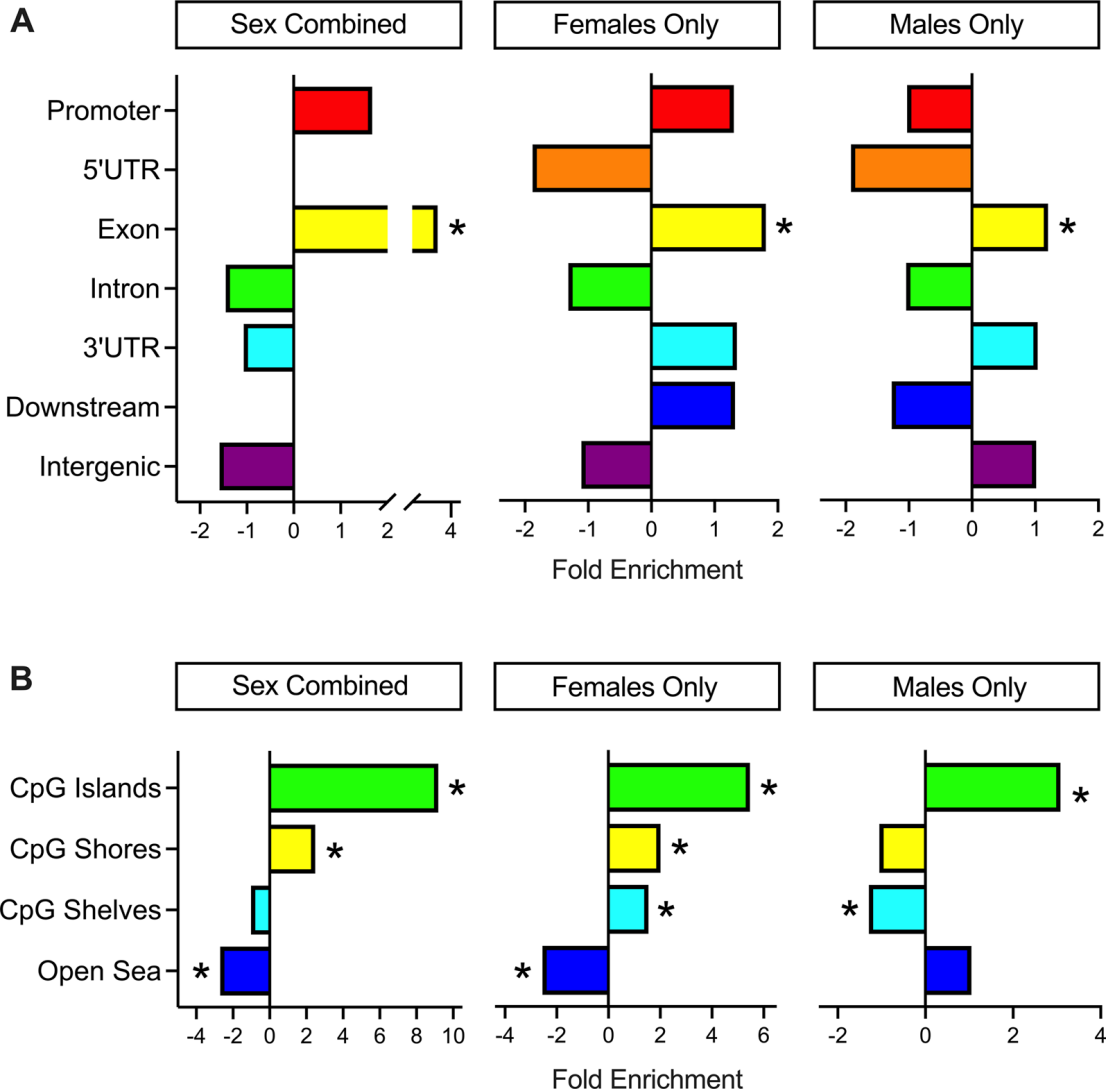
Annotation enrichments of CHD DMRs.** A** Genic and** B** CpG enrichments of all significant (*p* < 0.05) DMRs from Sex Combined, Females Only, and Males Only comparisons. DMRs were compared to background regions for each comparison, and significance was determined by the Fisher’s test and FDR correction. * = *q *< 0.05

CHD DMRs from Sex Combined, Females Only, and Males Only comparisons were analyzed for enrichment compared to background regions by distribution across chromosomes, genic and CpG contexts, histone marks, and chromatin states. In all three comparisons, DMRs were distributed throughout the genome (Additional file 2: Fig. S8), though Males Only DMRs showed significant enrichment (FDR < 0.05) on chromosomes 2, 4, 5, 8, 18, and 21, while Females Only DMRs showed nominal enrichment (unadjusted *p* < 0.05) on chromosomes 20, X, and 21 (Additional file 1: Table S16). There was significant positive enrichment in all comparisons for gene exons and CpG islands (Fig. 4) (Additional file 2: Figs. S9 and S10) (Additional file 1: Table S17), as well as the transcriptionally repressive H3K27me3 histone mark and bivalent enhancers and transcription start sites based on chromatin states (Additional file 2: Figs. S11–S16). Sex differences were also observed, with significant positive enrichment for CpG shelves in the Females Only comparison and significant negative enrichment in the Males Only comparison. The Females Only DMRs also showed enrichment for H3K4me3, associated with active/poised chromatin, while the Males Only DMRs showed enrichment for H3K9me3, another repressive mark (Additional file 2: Fig. S14). Hypomethylated regions showed overall stronger enrichment for histone marks and chromatin states compared to hypermethylated regions (Additional file 2: Figs. S15, S16).

### DS-CHD DMRs map to genes that are enriched for cardiac terms

**Fig. 5:**
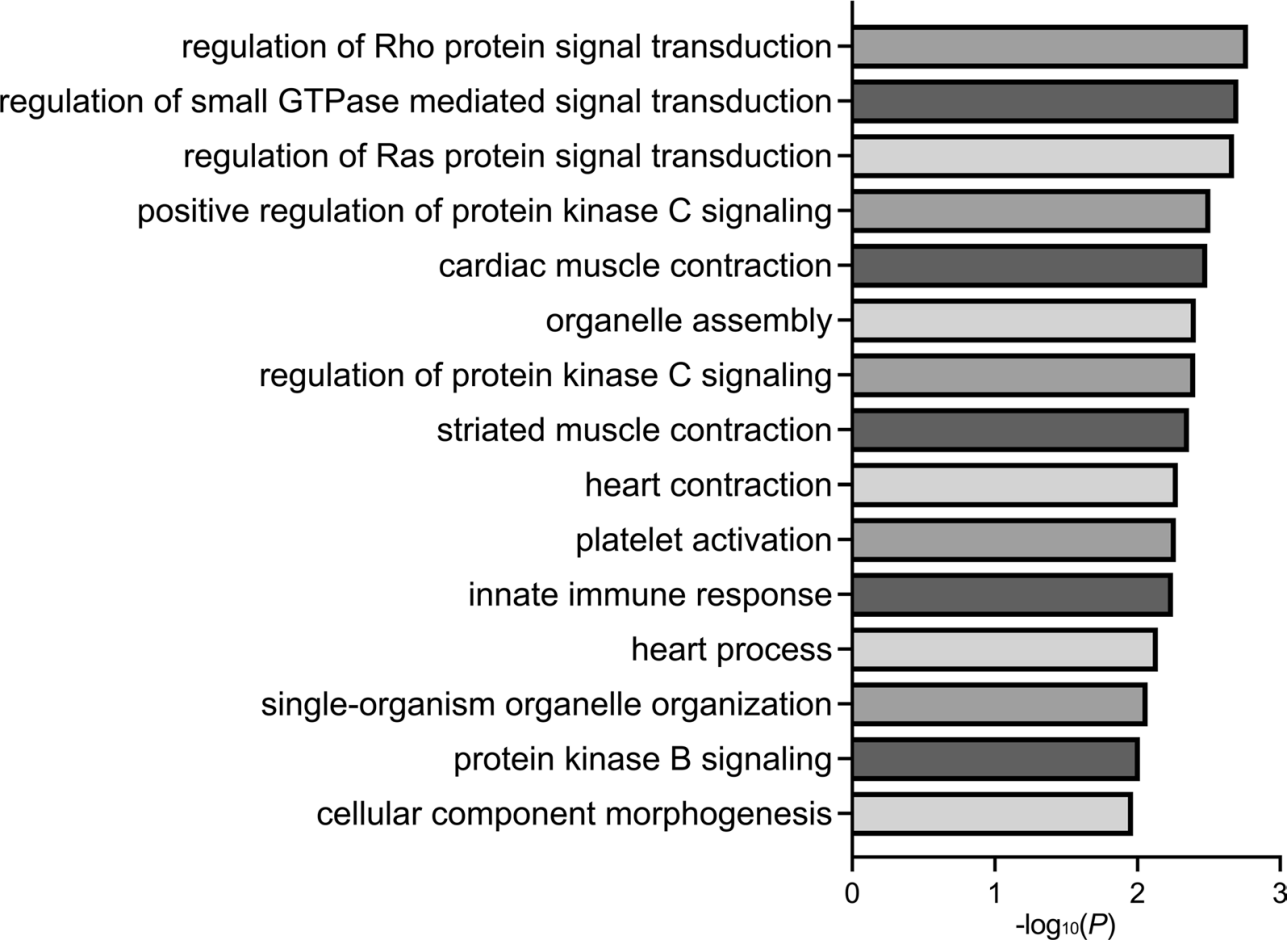
Gene ontology enrichments. Bar plot of the fifteen most significant GO enrichments for biological processes in DS-CHD versus DS non-CHD DMRs from the Sex Combined comparison

DMRs mapped to genes were analyzed for enrichment across Gene Ontology terms (*p* < 0.05) related to biological processes, cellular components, and molecular functions. All comparisons showed enrichment for heart-related terms, such as cardiac muscle contraction (Sex Combined) (Fig. 5), dorsal/ventral pattern formation, which includes formation of the embryonic heart tube (Females Only), and development of the septum primum, which divides the heart atrium into left and right and whose developmental failure can lead to AVSD (Males Only) (Additional files 1: Tables S18–S20, 2: Fig. S17). Genes contributing to the heart-related terms included *FGF12, PIK3CA, TNNI3, PDE4D*, *ACVR1, GATA4,* and others (Table 2). Enriched terms also included immune-related biological processes, such as platelet activation and innate immune response (Sex Combined) (Fig. 5).

**Table 2:** Heart-related biological processes identified from DMR Gene Ontology

Comparison	GO Term	Genes
Sex combined	Cardiac muscle contraction	*FGF12,PIK3CA,TNNI3*
	Striated muscle contraction	*FGF12,PIK3CA,TNNI3*
	Heart contraction	*FGF12,PIK3CA,TNNI3*
	Heart process	*FGF12,PIK3CA,TNNI3*
	Ductus arteriosus closure	*TFAP2B*
	Determination of dorsal/ventral asymmetry	*NBL1*
	Cellular response to erythropoietin	*CD40*
	Regulation of cardiac conduction	*NPR2,TNNI3*
	Muscle contraction	*FGF12,PIK3CA,TNNI3*
	Skeletal muscle contraction	*TNNI3*
Females only	Dorsal/ventral pattern formation	*FOXG1,GLI2,GREM2,INTU,MDFI,SMAD6,SUFU,TCTN1*
	Negative regulation of relaxation of cardiac muscle	*PDE4D*
	Negative regulation of heart contraction	*AGTR2,PDE4D*
	Adrenergic receptor signaling pathway involved in heart process	*PDE4D*
	Regulation of heart rate by chemical signal	*PDE4D*
	Regulation of relaxation of cardiac muscle	*PDE4D*
	Aorta development	*SMAD6,SUFU,TFAP2B,TGFB2*
	Regulation of ventricular cardiac muscle cell membrane repolarization	*ANK2,WDR1*
Males only	Septum primum development	*ACVR1,GATA4,GJA5,SOX4,TGFB2*
	Atrial septum primum morphogenesis	*ACVR1,GATA4,SOX4,TGFB2*
	Atrioventricular canal development	*CHD7,FOXN4,HAS2,PTPN11*
	Adult heart development	*ADRA1A,CHD7,HAND2,SCUBE1,TCAP*
	Artery smooth muscle contraction	*AGT,EDN1,EDN2,HTR2A,MKKS*
	Right ventricular compact myocardium morphogenesis	*CHD7*
	Atrial septum secundum morphogenesis	*GATA4*
	Positive regulation of heart rate	*ADRA1A,ADRB1,EDN1,EDN2,EDN3,KCNQ1,PDE4D,RYR2,SCN3B,TACR3,UTS2*
	Cardiac muscle hypertrophy	*AGT,GATA4,GATA6,HDAC4,KDM4A,LEP,PPP3CA,RYR2,TCAP,TIAM1,TTN*
	Positive regulation of heart contraction	*ADRA1A,ADRB1,EDN1,EDN2,EDN3,KCNQ1,PDE4D,RGS2,RYR2,SCN3B,TACR3,TGFB2,UTS2*
	Septum secundum development	*GATA4*
	Cardiac septum morphogenesis	*ACVR1,BMP4,BMP7,CHD7,FZD1,FZD2,GATA4,GATA6,GJA5,HES1,HEY1,HEYL,ISL1,JAG1,MSX2,NRP1,PARVA,PITX2,PROX1,RARB,SMAD6,SMAD7,SOX11,SOX4,TBX3,TGFB2,TGFBR2,ZFPM2*

### DS-CHD DMRs are also differentially methylated in DS versus typical development NDBS

**Fig. 6:**
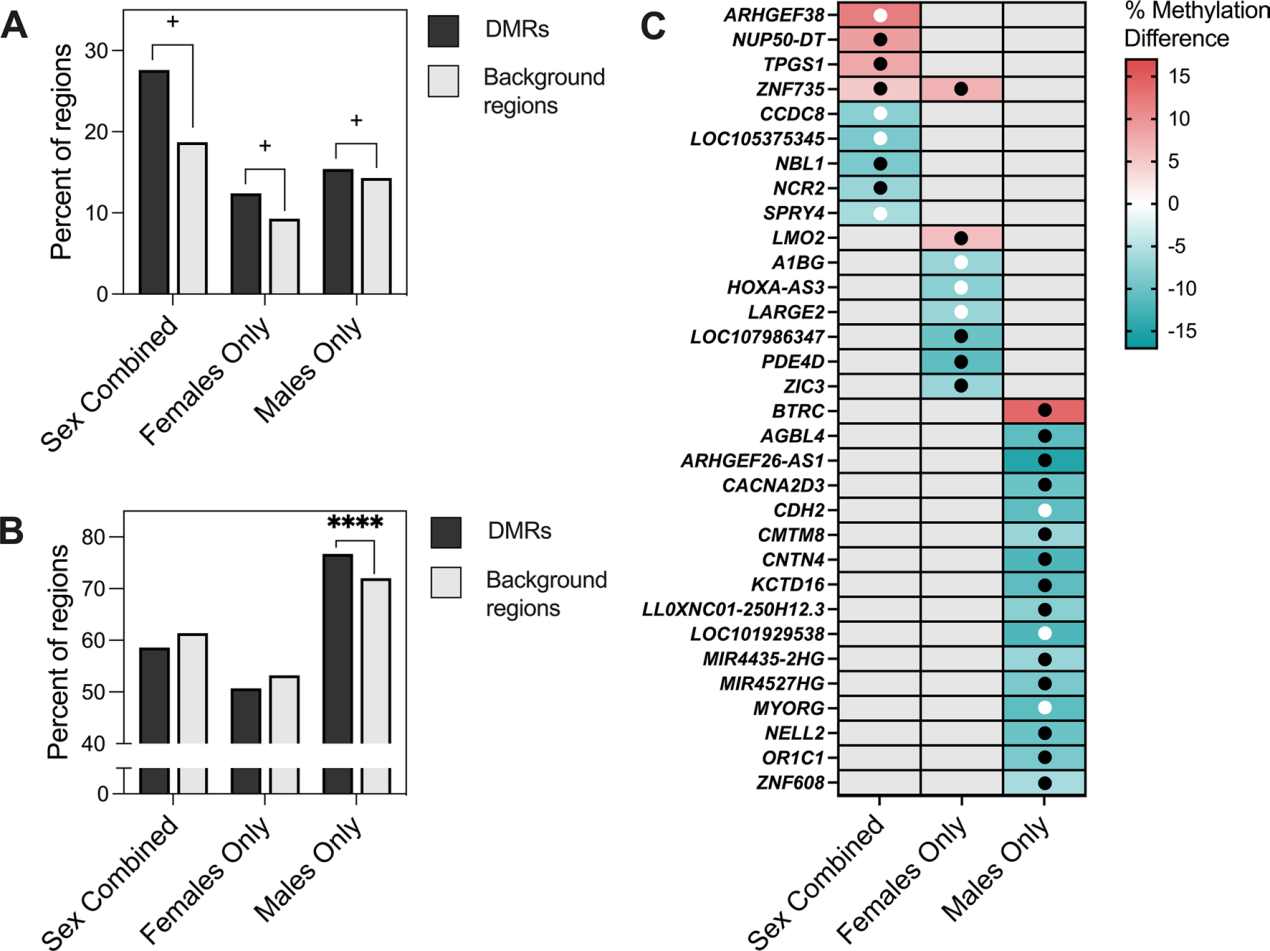
Comparison of DS-CHD DMRs with DS versus TD samples. **A** Percent of DS-CHD DMRs and background regions that were significantly differentially methylated in DS versus TD samples. *Z* test for two population proportions, Sex Combined (*z *= 1.7343, two-tailed *p *= 0.08364), Females Only (*z *= 1.93, two-tailed *p* = 0.0536), Males Only (*z *= 1.8808, two-tailed *p *= 0.0601). +  = *p* < 0.1. **B** Percent of DS-CHD DMRs that were methylated in same direction in DS versus TD as in DS-CHD versus DS non-CHD. *Z* test for two populations proportions, Sex Combined (*z *=  − 0.4274, two-tailed *p* = 0.6672), Females Only (*z *=  − 0.8936, two-tailed *p *= 0.37346), Males Only (*z *= 6.5357, two-tailed *p* < 0.00001). **** = *p* < 0.00001. **C** Heatmap showing DS-CHD DMRs that were significant (*q *< 0.05) in DS versus TD samples mapped to genes. Red indicates hypermethylation in CHD compared to non-CHD, while blue represents hypomethylation, with stronger shades representing a greater percent methylation difference and gray meaning that that DMR was not significant for that comparison. Black dots indicate that methylation is in the same direction for DS versus TD as DS-CHD versus DS non-CHD, while white dots indicate methylation is in the opposite direction

DS-CHD DMRs were tested for comparison in previously published DS versus TD NDBS WGBS samples [16] to evaluate the hypothesis that if DS-CHD is a more severe form of DS, CHD DMRs should be partially shared with DS versus TD DMRs (Table 3). Of the 58 Sex Combined CHD DMRs, 16 (27.6%) were significantly differentially methylated (*p* < 0.05) in DS versus TD samples (Additional file 1: Table S21), 9 of which (56.3%) were methylated in the same direction in DS versus TD samples as DS-CHD versus DS non-CHD samples. Of Females Only DMRs, 42/341 (12.3%) were significantly differentially methylated (*p* < 0.05) in DS versus TD, with 28 (66.7%) methylated in the same direction (Additional file 1: Table S22), and of Males Only DMRs, 602/3938 (15.3%) were significantly differentially methylated (*p* < 0.05) in DS versus TD, with 528 (87.7%) methylated in the same direction (Additional file 1: Table S23). These numbers decreased in a sensitivity analysis with the Males Only DMRs generated with five samples with nRBC > 0.2 removed, where 334/2454 (13.6%) covered DMRs were significantly (*p* < 0.05) differentially methylated in DS versus TD male samples, of which 248/334 (74.3%) were methylated in the same direction. For all three comparisons, there was a trend toward more CHD DMRs being significantly differentially methylated in DS versus TD samples compared to background regions (*z* test for two population proportions, Sex Combined *p* = 0.08364, Females Only *p* = 0.0536, Males Only *p* = 0.0601) (Fig. 6A). In Males Only, significantly more DMRs were methylated in the same direction in DS versus TD as DS-CHD versus DS non-CHD compared to background regions (*z* test for two population proportions, *p* < 0.00001), though this was not true for Sex Combined or Females Only CHD DMRs (Fig. 6B). Of DMRs that were significantly differentially methylated (*q* < 0.05) in DS versus TD samples, 5/9 (55.6%) Sex Combined, 6/8 (75%) Females Only, and 15/16 (93.85%) Males Only DMRs were hypomethylated in DS compared to TD samples. With the exception of an exon in *ZNF735*, which was significantly hypermethylated (*q* < 0.05) in both the Sex Combined and Females Only DS versus TD comparisons, all DMRs were specific to one comparison (Fig. 6C).

**Table 3:** Significance and direction of CHD DMRs and background regions in DS versus TD samples

	DMRs	Background regions
	*n* (%)	*n* (%)
*Sex combined*		
Total	58	5363
Omitted	0	28
*p* < 0.05	16 (27.6)	995 (18.7)
*p* ≥ 0.05	42 (72.4)	4340 (81.3)
Same direction	34 (58.6)	3274 (61.4)
Opposite direction	24 (41.4)	2061 (38.6)
*Females only*		
Total	341	11,998
Omitted	2	147
*p* < 0.05	42 (12.4)	1101 (9.3)
*p* ≥ 0.05	297 (87.6)	10,750 (90.7)
Same direction	172 (50.7)	6304 (53.2)
Opposite direction	167 (49.3)	5547 (46.8)
*Males only*		
Total	3938	127,819
Omitted	26	1413
*p* < 0.05	602 (15.4)	18,099 (14.3)
*p* ≥ 0.05	3310 (84.6)	108,307 (85.7)
Same direction	3001 (76.7)	90,954 (72.0)
Opposite direction	911 (23.3)	35,452 (28.0)

Same direction indicates methylation is in same direction (hypo or hyper) in DS versus TD as in DS-CHD versus non-CHD

## Discussion

This is the largest study to date to investigate epigenetic variation associated with CHDs in individuals with DS. Although non-syndromic CHDs have been widely studied, there has been relatively little research into the etiology and biomarkers of CHDs in individuals with DS, despite nearly half of the DS population presenting this phenotype [2–5]. To address this gap, we assessed DS-CHD methylation in DNA isolated from NDBSs, an understudied and accessible biospecimen that enables the analysis of epigenomic changes during the in utero and perinatal periods that are associated with phenotypic traits of interest. We confirmed the reproducibility of DNA extraction and bisulfite conversion from NDBSs by finding a high correlation (*r *= 0.9716) between global methylation levels from WGBS and EPIC array, which used different punches from the same blood spots.

Although newborn blood typically shows ~ 79–84% global CpG methylation from WGBS [16, 50], we found a range of 60.8–82.9% in our NDBS DNA. The samples with notably low WGBS global methylation also had low EPIC array beta values and passed other QC metrics, indicating that technical errors do not explain the result, although we cannot exclude non-biological causes. Previous studies have found a trend toward global hypomethylation in DS compared to TD NDBS [16], which may explain our findings. We further found that global methylation was lower in DS-CHD males compared to DS non-CHD males, though this relationship was not found in females. Global methylation was strongly negatively correlated with, and predicted by, nRBC proportion in both sexes. Although nRBCs typically constitute a very small proportion of nucleated cells, we found nRBC proportions ranged widely in both sexes and were evenly spread among CHD and non-CHD females but in males, 10/12 samples with nRBC > 1% were CHD positive. This discrepancy in the relationship between high nRBCs and CHD in males versus females likely explains the association between CHD and global hypomethylation in males and the lack of such association among females, given the strong effects of nRBC proportions on global methylation levels in DS newborns [17]. The etiology of the male-specific association between high nRBCs and CHD in newborns with DS remains to be determined.

Previous studies have reported high nRBC levels in DS newborns with pulmonary hypertension [51], as well as in hypoxic-related pregnancy situations, such as preeclampsia, maternal obesity and diabetes, maternal smoking, and prenatal exposure to infection [52–58]. Increased nRBC counts are thought to follow fetal hypoxemia through elevated erythropoietin (EPO), a hormone that stimulates production of erythrocytes (red blood cells) in an effort to increase oxygen delivery to tissues [59, 60]. Interestingly, EPO is higher in children with DS-CHD compared to non-syndromic CHD [61]. Because CHDs reduce cerebral oxygen [62] and may induce fetal hypoxemia [63], high nRBC proportions may be more common in individuals with CHDs and, in particular, in DS newborns with CHDs given the placental abnormalities seen in fetuses with trisomy 21 (PMID: 31683073). However, we could not confirm this hypothesis with our sample size. Moreover, nRBC proportions were estimated from DNA methylation array data, rather than using actual cell counts, and cell-type deconvolution in individuals with DS may be confounded by the presence of blast cells that are common in DS and not accounted for in the analysis [17]. Cell composition deconvoluted from DNA methylation arrays has been previously reported to be altered in DS blood compared to non-DS blood [64]. Further, we previously reported a positive relationship between high nRBC proportions in newborns with DS and the presence of somatic *GATA1* mutations, indicative of transient abnormal myelopoiesis (TAM) or silent TAM, and it is possible that the relationship between CHD and global hypomethylation in males may be confounded by this preleukemic condition [17]. Understanding of the complex relationships between CHDs in DS, global methylation, cell-type proportions, sex, and fetal hypoxia would benefit from further investigation.

In our WGBS regional analysis, we found over tenfold the number of CHD-associated DMRs in DS males than in females, and even fewer DMRs in the Sex Combined analysis. Reflecting our finding of global hypomethylation in CHD males, 96% of Males Only DMRs were hypomethylated, a pattern not seen in the Females Only or Sex Combined analyses. All DMRs were corrected for confounding factors including cell-type proportions, suggesting that nRBC levels were not fully responsible for the notable proportion of hypomethylated DMRs in males, although we cannot rule out residual confounding due to nRBCs or unmeasured traits related to nRBCs. Additionally, removing the five male samples with nRBC proportions > 20% resulted in 82% hypomethylated DMRs, suggesting that these five samples alone were not driving the signature of hypomethylation in DS-CHD males. Some DMRs from all comparisons were also differentially methylated in DS versus TD samples, and in males, a significantly higher proportion of DMRs were methylated in the same direction in DS versus TD and DS-CHD versus DS non-CHD compared to background regions. These results suggest that male DS patients with CHD may represent a more severe epigenomic signature than is observed for DS versus TD, although this may also reflect the higher nRBC proportions that have been reported in newborns with DS than in TD newborns (Muskens 2021). In contrast, female DS cases with CHD are somewhat epigenetically distinct from female and male DS cases without CHD. Response to hypoxia may play a role in these differences. DS newborns, even those without CHDs, experience more hypoxemia events than newborns with TD [65], and CHDs further induce fetal hypoxemia [63]. A wide variety of sex differences have been observed in response to hypoxia in both humans and animal models [66, 67], including differences in gene expression profiles of female versus male mice in cardiac adaptive responses to hypoxia [68]. These sex-specific responses to hypoxia may be reflected in the methylome, which is known to be influenced by gestational hypoxia [69] (reviewed in [70]). Identification of genes and pathways whose methylation and/or gene expression is altered in DS, CHDs, and hypoxia may help elucidate the sex specificity of molecular mechanisms related to DS-CHD.

Although we did not find any significant DMRs associated with DS-CHD after FDR correction, the nominally significant DMRs were enriched for genes implicated in cardiac processes, suggesting that at least some of the DMRs may reflect true epigenetic mechanisms associated with DS-CHD development. In particular, Males Only DMRs selected by machine learning feature selection were able to distinguish CHD from non-CHD samples and frequently mapped to genes associated with CHDs or cardiomyopathies, including *FUNDC1* [71], *ETV5* [72, 73], *SYT9* [74], *CAMTA1* [75], *GRIA4* [76], and *IGF1R* [77–80]. Additionally, DMRs that contributed to enrichment for heart-related gene ontology terms included *TNNI3*, a cardiac-specific gene that codes for cardiac troponin I, whose absence leads to severe pediatric cardiomyopathy [81], and *GATA4,* which encodes a member of the GATA family of zinc finger transcription factors, are essential for mammalian cardiac development, and whose sequence variants have been identified in individuals with CHDs [82]. Whether the differential methylation in the genes we identified plays an etiologic role or reflects epigenomic effects downstream of the development of CHDs remains to be determined.

While this is, to our knowledge, the largest DNA methylation study of CHDs in DS, our sample size of 86 DS newborns may still have limited our ability to detect genome-wide significant (*q* < 0.05) DMRs. Additionally, only around half of DMRs in all comparisons were methylated in the same direction in the discovery and replication groups, potentially due to the very small sample sizes and absence of confounding variable data to use for correction in the replication group, as well as high interindividual variation in methylation. The genes to which our DMRs mapped did not heavily coincide with those identified in previous epigenetic studies of DS-CHD [83, 84], likely because those studies included small numbers of DS subjects, used non-NDBS biospecimens assayed with array-based methods, which do not have good coverage over the regions we detected using WGBS, and did not account for cell-type heterogeneity. One exception to this is that we identified a DMR in the Males Only comparison that mapped to *SHC3*, a gene that was differentially expressed in DS individuals with an endocardial cushion CHD [83]. The DS field would benefit from further studies into the etiology and biomarkers of phenotypes common in the DS population, including CHDs.

## Conclusions

Overall, this study presents the largest investigation of epigenetic variation associated with CHDs in individuals with DS. We identified sex-specific global and regional methylation differences in DS-CHD versus DS non-CHD newborns. Specifically, in males we found that newborns with DS-CHD were globally hypomethylated compared to DS newborns without CHD, a finding that appeared to be driven by differences in nRBC proportions between the two groups. At the regional level, the majority of CHD DMRs identified by sex stratification did not overlap by genomic coordinates, suggesting sex differences in the molecular signature of CHDs in DS. Gene ontology analysis of DMRs from both sexes revealed enrichment in pathways related to the heart, and some DS-CHD DMRs were also differentially methylated in DS versus TD samples. Our results provide insight into the development of CHDs in newborns with DS, pointing to sex-specific differences that warrant further investigation, and suggest that DNA methylation may serve as a useful biomarker for investigating the variability of clinical features within the genetic disorder of DS.

### Supplementary Information

**Additional file 1: Table S1.** Study characteristics: description of sample traits included in study. **Table S2**. Sample traits: values of traits for each sample. **Table S3**. Welch’s *t* test for differences in sample traits in DS-CHD versus DS non-CHD samples. **Table S4.** Pearson correlation coefficients and *p* values (unadjusted and adjusted) for sample traits. **Table S5**. EPIC array beta values. **Table S6**. Logistic regression for CHD and linear regression for global methylation. **Table S7**. Annotated Sex Combined DMRs (adjusted for sex, age of blood collection, cell types). **Table S8**. Annotated Females Only DMRs (adjusted for age of blood collection, cell types). **Table S9**. Annotated Males Only DMRs (adjusted for age of blood collection, cell types). **Table S10**. Annotated Males Only DMRs (adjusted for age of blood collection, cell types) Sensitivity Analysis (5 samples with nRBC > 20% removed). **Table S11**. Machine Learning DMRs. **Table S12**. Smoothed methylation of Sex-combined DMRs in replication dataset. **Table S13**. Smoothed methylation of Females Only DMRs in replication dataset. **Table S14**. Smoothed methylation of Males Only DMRs in replication dataset. **Table S15**. Stats from permutation testing of DMR overlaps from Sex Combined, Females Only, and Males Only comparisons. **Table S16**. Chromosome location enrichments of Sex Combined, Females Only, and Males Only DMRs. **Table S17**. CpG and Genic enrichments for Sex Combined, Females Only, and Males Only comparisons. **Table S18**. GREAT gene ontology enrichments for Sex-Combined DMRs compared to background regions. **Table S19**. GREAT gene ontology enrichments for Females Only DMRs compared to background regions. **Table S20**. GREAT gene ontology enrichments for Males Only DMRs compared to background regions. **Table S21**. Smoothed methylation of Sex-combined DMRs in DSvTD dataset. **Table S22**. Smoothed methylation of Females Only DMRs in DSvTD dataset. **Table S23**. Smoothed methylation of Males Only DMRs in DSvTD dataset.

**Additional file 2: Fig. S1.** **A** Average WGBS Bismark coverage for 86 samples included in analysis (minimum 3.696, maximum 7.561, median 3.967, mean 4.088) and **B** Total number of CpGs remaining for the analysis following minimum coverage and sample inclusion cutoffs. **Fig. S2.** Sample trait correlations in **A** all samples, **B** females only, and **C** males only by Pearson correlation. **Fig. S3.** Global methylation values from WGBS are highly correlated with those from the EPIC array. Pearson’s *r* = 0.9716, *p *< 0.0001. **Fig. S4.** Violin plots with all points shown for **A** global methylation and **B** nRBC proportion in females and males with DS CHD (Yes) and DS non-CHD (No). *p < 0.05 in Welch’s unequal variances *t* test. **Fig. S5.** Hierarchical clustering heatmap of the machine learning feature selection analysis of the consensus DMRs from **A** Sex Combined and **B** Females Only comparisons. **Fig. S6**. Sex specificity of DMRs. Principal component analysis of smoothed methylation values over DMRs from the **A** Females Only comparison in male samples and **B** Males Only comparison in female samples. All DMRs from the Females Only comparison were used and the top 1000 most significant DMRs in male samples from the Males Only comparison were used. **Fig. S7.** Sex Combined DMRs do not separate by sex. PCA analysis using the smoothed methylation values of all DMRs from the Sex Combined comparison, colored by sex. **Fig. S8.** Manhattan plots of tested background regions for the **A** Sex-combined, **B** Females Only, and **C** Males Only comparisons. The density heatmap indicates the number of DMRs in 1 Mb bins and the line in the Manhattan plot indicates non-adjusted *p* = 0.05. **Fig. S9.** Genic annotation enrichments of all DMRs, hypermethylated DMRs, and hypomethylated DMRs in **A** Sex-combined, **B** Females Only, and **C** Males Only comparisons. *q < 0.05. **Fig. S10.** CpG annotation enrichments of all DMRs, hypermethylated DMRs, and hypomethylated DMRs in **A** Sex-combined, **B** Females Only, and **C** Males Only comparisons. *q < 0.05. **Fig. S11.** Heatmap of odds ratios for roadmap epigenomics 127 reference epigenomics 127 reference epigenomes core chromatin state enrichments for all DMRs for DS CHD versus DS non-CHD in **A** Sex-combined comparison **B** Females Only comparison **C** Males Only comparison. **Fig. S12.** Heatmap of odds ratios for roadmap epigenomics 127 reference epigenomes core chromatin state enrichments for hypermethylated DMRs for DS CHD versus DS non-CHD in **A** Sex-combined comparison **B** Females Only comparison **C** Males Only comparison. **Fig. S13.** Heatmap of odds ratios for roadmap epigenomics 127 reference epigenomes core chromatin state enrichments for hypomethylated DMRs for DS CHD versus DS non-CHD in **A** Sex-combined comparison **B** Females Only comparison **C** Males Only comparison. **Fig. S14.** Heatmap of odds ratios for roadmap epigenomics 127 reference epigenomes core histone modifications enrichments for all DMRs for DS CHD versus DS non-CHD in **A** Sex-combined comparison **B** Females Only comparison **C** Males Only comparison. **Fig. S15.** Heatmap of odds ratios for roadmap epigenomics 127 reference epigenomes core histone modifications enrichments for hypermethylated DMRs for DS CHD versus DS non-CHD in **A** Sex-combined comparison **B** Females Only comparison **C** Males Only comparison. **Fig. S16.** Heatmap of odds ratios for roadmap epigenomics 127 reference epigenomes core histone modifications enrichments for hypomethylated DMRs for DS CHD versus DS non-CHD in **A** Sex-combined comparison **B** Females Only comparison **C** Males Only comparison. **Fig. S17.** Gene ontology enrichments. Bar plot of the fifteen most significant GO enrichments for biological processes in DS-CHD versus DS non-CHD DMRs from the **A** Females Only comparison and **B** Males Only comparison.

## Data Availability

This study used biospecimens from the California Biobank Program. Any uploading of genomic data (including genome‐wide DNA methylation data) and/or sharing of these biospecimens or individual data derived from these biospecimens has been determined to violate the statutory scheme of the California Health and Safety Code Sects. 124980(j), 124991(b), (g), (h), and 103850 (a) and (d), which protect the confidential nature of biospecimens and individual data derived from biospecimens. Should we be contacted regarding individual-level data contributing to the findings reported in this study, inquiries will be directed to the California Department of Public Health Institutional Review Board to establish an approved protocol to utilize the data, which cannot otherwise be shared peer-to-peer. *Code Available* at https://github.com/juliamouat/DownSyndrome_CongenitalHeartDefect_DNAmethylation.

## Abbreviations

DS: Down syndrome
CHD: Congenital heart defect
AVSD: Atrioventricular septal defect
WGBS: Whole-genome bisulfite sequencing
NDBS: Newborn dried blood spot
TD: Typical development
DMR: Differentially methylated region
QC: Quality control
nRBC: Nucleated red blood cell
PCA: Principal component analysis
EPO: Erythropoietin
TAM: Transient abnormal myelopoiesis

## References

[1] Antonarakis SE, Skotko BG, Rafii MS, Strydom A, Pape SE, Bianchi DW (2020). Down syndrome. Nat Rev Dis Primers.

[2] Dobosz A, Bik-Multanowski M (2019). Long-term trends in the prevalence of congenital heart defects in patients with down syndrome in Southern Poland. Dev Period Med.

[3] Irving CA, Chaudhari MP (2012). Cardiovascular abnormalities in Down’s syndrome: spectrum, management and survival over 22 years. Arch Dis Child.

[4] Laursen HB (1976). Congenital heart disease in Down’s syndrome. Br Heart J.

[5] Weijerman ME, van Furth AM, Vonk Noordegraaf A, van Wouwe JP, Broers CJM, Gemke RJBJ (2008). Prevalence, neonatal characteristics, and first-year mortality of down syndrome: a national study. J Pediatr.

[6] Mai CT, Isenburg JL, Canfield MA, Meyer RE, Correa A, Alverson CJ (2019). National population-based estimates for major birth defects, 2010–2014. Birth Defects Res.

[7] Korbel JO, Tirosh-Wagner T, Urban AE, Chen XN, Kasowski M, Dai L (2009). The genetic architecture of down syndrome phenotypes revealed by high-resolution analysis of human segmental trisomies. Proc Natl Acad Sci U S A.

[8] Ackerman C, Locke AE, Feingold E, Reshey B, Espana K, Thusberg J (2012). An excess of deleterious variants in VEGF-A pathway genes in down-syndrome-associated atrioventricular septal defects. Am J Hum Genet.

[9] Ramachandran D, Mulle JG, Locke AE, Bean LJH, Rosser TC, Bose P (2015). Contribution of copy number variation to down syndrome-associated atrioventricular septal defects. Genet Med.

[10] Ramachandran D, Zeng Z, Locke AE, Mulle JG, Bean LJH, Rosser TC (2015). Genome-wide association study of down syndrome-associated atrioventricular septal defects. G3.

[11] Rambo-Martin BL, Mulle JG, Cutler DJ, Bean LJH, Rosser TC, Dooley KJ (2017). Analysis of copy number variants on chromosome 21 in down syndrome-associated congenital heart defects. G3.

[12] Sailani MR, Makrythanasis P, Valsesia A, Santoni FA, Deutsch S, Popadin K (2013). The complex SNP and CNV genetic architecture of the increased risk of congenital heart defects in Down syndrome. Genome Res.

[13] Trevino CE, Holleman AM, Corbitt H, Maslen CL, Rosser TC, Cutler DJ (2020). Identifying genetic factors that contribute to the increased risk of congenital heart defects in infants with Down syndrome. Sci Rep.

[14] Cao J, Wu Q, Huang Y, Wang L, Su Z, Ye H (2021). The role of DNA methylation in syndromic and non-syndromic congenital heart disease. Clin Epigenet.

[15] Vecoli C, Pulignani S, Foffa I, Andreassi MG (2014). Congenital heart disease: the crossroads of genetics. Epigenet Environ Curr Genom.

[16] Laufer BI, Hwang H, Jianu JM, Mordaunt CE, Korf IF, Hertz-Picciotto I (2020). Low-pass whole genome bisulfite sequencing of neonatal dried blood spots identifies a role for RUNX1 in down syndrome DNA methylation profiles. Hum Mol Genet.

[17] Muskens IS, Li S, Jackson T, Elliot N, Hansen HM, Myint SS (2021). The genome-wide impact of trisomy 21 on DNA methylation and its implications for hematopoiesis. Nat Commun.

[18] Croen LA, Shaw GM, Jensvold NG, Harris JA (1991). Birth defects monitoring in California: a resource for epidemiological research. Paediatr Perinat Epidemiol.

[19] Laufer BI. FASTQ_Me [Internet]. 2020 [cited 2023 Feb 28]. Available from: https://github.com/ben-laufer/FASTQ_Me

[20] Laufer BI. CpG_Me [Internet]. 2022 [cited 2022 Jan 20]. Available from: https://github.com/ben-laufer/CpG_Me

[21] Ewels P, Magnusson M, Lundin S, Käller M (2016). MultiQC: summarize analysis results for multiple tools and samples in a single report. Bioinformatics.

[22] Krueger F, Andrews SR (2011). Bismark: a flexible aligner and methylation caller for Bisulfite-Seq applications. Bioinformatics.

[23] Laufer BI, Neier K, Valenzuela AE, Yasui DH, Schmidt RJ, Lein PJ (2022). Placenta and fetal brain share a neurodevelopmental disorder DNA methylation profile in a mouse model of prenatal PCB exposure. Cell Rep.

[24] Martin M (2011). Cutadapt removes adapter sequences from high-throughput sequencing reads. EMBnet J.

[25] Hovestadt V, Zapatka M. conumee: Enhanced copy-number variation analysis using Illumina DNA methylation arrays [Internet]. Available from: http://bioconductor.org/packages/conumee/10.1093/bioinformatics/btae029 PMC10868300 38244574

[26] Du P, Zhang X, Huang CC, Jafari N, Kibbe WA, Hou L (2010). Comparison of beta-value and M-value methods for quantifying methylation levels by microarray analysis. BMC Bioinf.

[27] Pidsley R, Zotenko E, Peters TJ, Lawrence MG, Risbridger GP, Molloy P (2016). Critical evaluation of the Illumina MethylationEPIC BeadChip microarray for whole-genome DNA methylation profiling. Genome Biol.

[28] Xu Z, Xie C, Taylor JA, Niu L (2020). ipDMR: identification of differentially methylated regions with interval P-values. Bioinformatics.

[29] Koestler DC, Jones MJ, Usset J, Christensen BC, Butler RA, Kobor MS (2016). Improving cell mixture deconvolution by identifying optimal DNA methylation libraries (IDOL). BMC Bioinf.

[30] Salas LA, Koestler DC. FlowSorted.Blood.EPIC: Illumina EPIC data on immunomagnetic sorted peripheral adult blood cells [Internet]. 2023. Available from: https://github.com/immunomethylomics/FlowSorted.Blood.EPIC

[31] Salas LA, Gervin K, Jones MC. FlowSorted.CordBloodCombined.450k: Illumina 450k/EPIC data on FACS and MACS umbilical blood cells [Internet]. 2023. Available from: https://github.com/immunomethylomics/FlowSorted.CordBloodCombined.450k

[32] Harrell FE Jr, with contributions from Charles Dupont and many others. Hmisc: Harrell miscellaneous [Internet]. 2019. Available from: https://CRAN.R-project.org/package=Hmisc

[33] Revelle W. psych: procedures for psychological, psychometric, and personality research [Internet]. Northwestern University, Evanston, Illinois; 2022. Available from: https://CRAN.R-project.org/package=psych

[34] Mordaunt CE, Mouat JS, Schmidt RJ, LaSalle JM (2022). Comethyl: a network-based methylome approach to investigate the multivariate nature of health and disease. Brief Bioinf.

[35] Hansen KD, Langmead B, Irizarry RA (2012). BSmooth: from whole genome bisulfite sequencing reads to differentially methylated regions. Genome Biol.

[36] Korthauer K, Chakraborty S, Benjamini Y, Irizarry RA (2019). Detection and accurate false discovery rate control of differentially methylated regions from whole genome bisulfite sequencing. Biostatistics.

[37] Haftorn KL, Denault WRP, Lee Y, Page CM, Romanowska J, Lyle R (2023). Nucleated red blood cells explain most of the association between DNA methylation and gestational age. Commun Biol.

[38] Kursa MB, Rudnicki WR (2010). Feature selection with the boruta package. J Stat Softw.

[39] Das P, Roychowdhury A, Das S, Roychoudhury S, Tripathy S. sigFeature: novel significant feature selection method for classification of gene expression data using support vector machine and t statistic. Front Genet [Internet]. 2020 [cited 2023 Feb 13];11. Available from: https://www.frontiersin.org/articles/10.3389/fgene.2020.00247 10.3389/fgene.2020.00247 PMC7169426 32346383

[40] Lawrence M, Gentleman R, Carey V (2009). rtracklayer: an R package for interfacing with genome browsers. Bioinformatics.

[41] Lawrence M, Huber W, Pagès H, Aboyoun P, Carlson M, Gentleman R (2013). Software for computing and annotating genomic ranges. PLoS Comput Biol.

[42] Chen H, Boutros PC (2011). VennDiagram: a package for the generation of highly-customizable Venn and Euler diagrams in R. BMC Bioinf.

[43] Gel B, Díez-Villanueva A, Serra E, Buschbeck M, Peinado MA, Malinverni R (2016). regioneR: an R/Bioconductor package for the association analysis of genomic regions based on permutation tests. Bioinformatics.

[44] Huang DW, Sherman BT, Lempicki RA (2009). Systematic and integrative analysis of large gene lists using DAVID bioinformatics resources. Nat Protoc.

[45] Huang DW, Sherman BT, Lempicki RA (2009). Bioinformatics enrichment tools: paths toward the comprehensive functional analysis of large gene lists. Nucleic Acids Res.

[46] McLean CY, Bristor D, Hiller M, Clarke SL, Schaar BT, Lowe CB (2010). GREAT improves functional interpretation of cis-regulatory regions. Nat Biotechnol.

[47] U.S. Census Bureau. U.S. Census Bureau QuickFacts: California [Internet]. [cited 2023 Sep 22]. Available from: https://www.census.gov/quickfacts/fact/table/CA/PST045222

[48] de Goede OM, Lavoie PM, Robinson WP (2016). Characterizing the hypomethylated DNA methylation profile of nucleated red blood cells from cord blood. Epigenomics.

[49] Salas LA, Zhang Z, Koestler DC, Butler RA, Hansen HM, Molinaro AM (2022). Enhanced cell deconvolution of peripheral blood using DNA methylation for high-resolution immune profiling. Nat Commun.

[50] Mordaunt CE, Jianu JM, Laufer B, Zhu Y, Dunaway KW, Bakulski KM, et al. Cord blood DNA methylome in newborns later diagnosed with autism spectrum disorder reflects early dysregulation of neurodevelopmental and X-linked genes [Internet]. Genomics; 2019 Nov [cited 2020 Apr 15]. Available from: http://biorxiv.org/lookup/doi/10.1101/850529 10.1186/s13073-020-00785-8 PMC7559201 33054850

[51] Nitzan I, Kasirer Y, Mimouni FB, Fink D, Wasserteil N, Hammerman C (2019). Elevated nucleated red blood cells in neonates with down syndrome and pulmonary hypertension. J Pediatr.

[52] Aali BS, Malekpour R, Sedig F, Safa A (2007). Comparison of maternal and cord blood nucleated red blood cell count between pre-eclamptic and healthy women. J Obstet Gynaecol Res.

[53] Baschat AA, Gungor S, Kush ML, Berg C, Gembruch U, Harman CR (2007). Nucleated red blood cell counts in the first week of life: a critical appraisal of relationships with perinatal outcome in preterm growth-restricted neonates. Am J Obstet Gynecol.

[54] de Goede OM, Razzaghian HR, Price EM, Jones MJ, Kobor MS, Robinson WP (2015). Nucleated red blood cells impact DNA methylation and expression analyses of cord blood hematopoietic cells. Clin Epigenet.

[55] Hermansen M (2001). Nucleated red blood cells in the fetus and newborn. Arch Dis Child Fetal Neonatal Ed.

[56] Redline RW (2008). Elevated circulating fetal nucleated red blood cells and placental pathology in term infants who develop cerebral palsy. Hum Pathol.

[57] Yeruchimovich M, Dollberg S, Green DW, Mimouni FB (1999). Nucleated red blood cells in infants of smoking mothers. Obstet Gynecol.

[58] Yeruchimovich M, Mimouni FB, Green DW, Dollberg S (2000). Nucleated red blood cells in healthy infants of women with gestational diabetes. Obstet Gynecol.

[59] Bedrick AD (2014). Nucleated red blood cells and fetal hypoxia: a biologic marker whose ‘timing’ has come?. J Perinatol.

[60] Teramo KA, Widness JA (2009). Increased fetal plasma and amniotic fluid erythropoietin concentrations: markers of intrauterine hypoxia. Neonatology.

[61] Zakharchenko L (2022). Infants with down syndrome and congenital heart disease have altered peri-operative immune responses. Pediatr Res.

[62] Morton PD, Korotcova L, Lewis BK, Bhuvanendran S, Ramachandra SD, Zurakowski D (2017). Abnormal neurogenesis and cortical growth in congenital heart disease. Sci Transl Med..

[63] Peyvandi S, Xu D, Wang Y, Hogan W, Moon-Grady A, Barkovich AJ (2021). Fetal cerebral oxygenation is impaired in congenital heart disease and shows variable response to maternal hyperoxia. J Am Heart Assoc.

[64] Zhang Z, Stolrow HG, Christensen BC, Salas LA (2023). Down syndrome altered cell composition in blood, brain, and buccal swab samples profiled by DNA-methylation-based cell-type deconvolution. Cells.

[65] Krahn KN, Nagraj VP, McCulloch MA, Zimmet AM, Fairchild KD (2021). Hypoxemia in infants with trisomy 21 in the neonatal intensive care unit. J Perinatol.

[66] Horiuchi M, Kirihara Y, Fukuoka Y, Pontzer H (2019). Sex differences in respiratory and circulatory cost during hypoxic walking: potential impact on oxygen saturation. Sci Rep.

[67] Mayoral SR, Omar G, Penn AA (2009). Sex differences in a hypoxia model of preterm brain damage. Pediatr Res.

[68] Bohuslavová R, Kolář F, Kuthanová L, Neckář J, Tichopád A, Pavlinkova G (2010). Gene expression profiling of sex differences in HIF1-dependent adaptive cardiac responses to chronic hypoxia. J Appl Physiol.

[69] Zhu Y, Gomez JA, Laufer BI, Mordaunt CE, Mouat JS, Soto DC (2022). Placental methylome reveals a 22q13.33 brain regulatory gene locus associated with autism. Genome Biol.

[70] Ma Q, Xiong F, Zhang L (2014). Gestational hypoxia and epigenetic programming of brain development disorders. Drug Discov Today.

[71] Liu L, Li Y, Chen Q. The emerging role of FUNDC1-mediated mitophagy in cardiovascular diseases. Front Physiol [Internet]. 2021 [cited 2023 Mar 2];12. Available from: https://www.frontiersin.org/articles/10.3389/fphys.2021.807654 10.3389/fphys.2021.807654 PMC8718682 34975548

[72] Liu Y, Lu P, Wang Y, Morrow BE, Zhou B, Zheng D (2019). Spatiotemporal gene coexpression and regulation in mouse cardiomyocytes of early cardiac morphogenesis. J Am Heart Assoc.

[73] Tan WLW, Anene-Nzelu CG, Wong E, Lee CJM, Tan HS, Tang SJ (2020). Epigenomes of human hearts reveal new genetic variants relevant for cardiac disease and phenotype. Circ Res.

[74] Xie HH, Li J, Li PQ, Zhang AA, Li Y, Wang YZ (2017). A genetic variant in a homocysteine metabolic gene that increases the risk of congenital cardiac septal defects in Han Chinese populations. IUBMB Life.

[75] Song K, Backs J, McAnally J, Qi X, Gerard RD, Richardson JA (2006). The transcriptional coactivator CAMTA2 stimulates cardiac growth by opposing class II histone deacetylases. Cell.

[76] Izarzugaza JMG, Ellesøe SG, Doganli C, Ehlers NS, Dalgaard MD, Audain E (2020). Systems genetics analysis identifies calcium-signaling defects as novel cause of congenital heart disease. Genome Med.

[77] Benbouchta Y, De Leeuw N, Amasdl S, Sbiti A, Smeets D, Sadki K (2021). 15q26 deletion in a patient with congenital heart defect, growth restriction and intellectual disability: case report and literature review. Ital J Pediatr.

[78] González-Guerra JL, Castilla-Cortazar I, Aguirre GA, Muñoz Ú, Martín-Estal I, Ávila-Gallego E (2017). Partial IGF-1 deficiency is sufficient to reduce heart contractibility, angiotensin II sensibility, and alter gene expression of structural and functional cardiac proteins. PLoS ONE.

[79] Huynh K, McMullen JR, Julius TL, Tan JW, Love JE, Cemerlang N (2010). Cardiac-specific IGF-1 receptor transgenic expression protects against cardiac fibrosis and diastolic dysfunction in a mouse model of diabetic cardiomyopathy. Diabetes.

[80] Ock S, Lee WS, Ahn J, Kim HM, Kang H, Kim HS (2016). Deletion of IGF-1 receptors in cardiomyocytes attenuates cardiac aging in male mice. Endocrinology.

[81] Kühnisch J, Herbst C, Al-Wakeel-Marquard N, Dartsch J, Holtgrewe M, Baban A (2019). Targeted panel sequencing in pediatric primary cardiomyopathy supports a critical role of TNNI3. Clin Genet.

[82] Tomita-Mitchell A, Maslen CL, Morris CD, Garg V, Goldmuntz E (2007). GATA4 sequence variants in patients with congenital heart disease. J Med Genet.

[83] Dobosz A, Grabowska A, Bik-Multanowski M (2019). Hypermethylation of NRG1 gene correlates with the presence of heart defects in Down’s syndrome. J Genet.

[84] Serra-Juhé C, Cuscó I, Homs A, Flores R, Torán N, Pérez-Jurado LA (2015). DNA methylation abnormalities in congenital heart disease. Epigenetics.

